# Improvements in Operative Dentistry. What Is Demanded of Us and What We Should Exact

**Published:** 1874-06

**Authors:** Wm. F. Thompson.


### DENTAL REGISTER.

VOL. XXVIII.] JUNE, 1874. [No. 6

### IMPROVEMENTS IN OPERATIVE DENTISTRY, WHAT IS DEMANDED OF US, AND WHAT WE SHOULD EXACT.

*Read before the Oregon State Dental Society, March 25th,* 1874.

BY WM. F. THOMPSON., D. D. S.

*Gentlemen:* It is with pleasure, that on this, our first anniversary, I meet with you again, and by the number present, am led to believe, that you are earnestly working to advance a calling that is made proportionately respectable as are its representatives professional in their acts. To figuratively speak: the professional timber of our young state will, and does, compare favorably with that of the Atlantic states.

Our neglected pines, though not so handsome as the cultivated maple, make up in magnificent proportions, what they lack in grace and beauty. One year ago to-day, Oregon was represented by individual dentists only. She boasted no state or local society, but our organization, now numbers seventeen members; as good average material as can be found in any country; and I, as one of you, hold sacred the privilege of enjoying these pleasant reunions.

The subject assigned me, by our most worthy presiding officer, makes me feel, that he, like the rest of us, will occasionally make mistakes, and should the expectations of my victimized listeners be dashed, I charge them, that to the author of their woes, must they lay the blame.

However, I accept my apportioned part as a duty, and in entering upon the discharge of said duty, the primary object, in view, will be to advocate our advancement to the highest point of excellence.

But before commencing, I desire to preface, by making a few remarks upon a self-appointed subject, viz: our *social* and *professional advancement.* Through the medium of our society, an interest has been promoted, to foster on the part of individual members, that desire for self-culture, which is the marked characteristic of this age, and to this end, will our united efforts stimulate us, to greater deeds, than could otherwise be accomplished. While we should proudly maintain for our own profession a position second to none other, we must also comprehend the importance of making available every opportunity to fit ourselves for the position we expect to occupy.

With these self-evident facts so plainly attested, how important it is that every city and town, of any size, should have its local dental society. The successful prosecution of the present system of dental education demands that these associations should be liberally supported, and that there should be harmony and concert of action among them; whoever attempts to destroy that harmony is an enemy to our cause, and should be cast out from among us. By association, men grow more bold in advancing their ideas, and express themselves more fluently as speakers; the diffident gain confidence in self, while the assuming and over-confident meet those sharp projections and antagonistic forces, which compell them to occupy their true position. Language, to us, is a vehicle for the conveyance of thought, and the more perfectly it is understood by a writer, the more instructive and attractive he becomes.

But while we pay a proper tribute to language, we can not fail to justly acknowledge the homage that is due those nobler traits—when not debased—which makes a man the endorser of his words, for by his acts, we judge the sincerity of his intentions. Let us look a little closer at our representative dentist, his position, and what is expected of him.

The man who engages in dentistry should do so with a full knowledge of the responsibility, patience, toil and anxiety it necessarily involves.

He must remember that he is not at work on inanimate material, but is operating upon extremely sensitive tissue; that the health and comfort of his patient may be affected for good or evil through life, by his skillful or unskillful operations.

The conscientious dentist, will most sacredly observe the rights and privileges which belong to his competitors in business, his patrons and himself. He is fully aware that to keep up with the rapid advance that is being continually made, he must study hard, that he may be master of his position. In justice, it may be said, that dentistry was practiced—crudely, we must admit—dating back into the dark ages, but to the fertile brain and skillful fingers of the American dentist rightfully belongs, and is assigned, the honor of almost every improvement belonging to our profession. To him, is justly due the patient investigation, revising and perfecting, until ours is no longer an unpretentious calling, defended and supported by a few, but has grown in strength, until it stands out boldly for, and stoutly demands a recognition of its rights.

From comparative obscurity, it has been raised to a position attended with many substantial advantages, and this has been done by a few self sacrificing men, who have chosen to sustain a uniform and strictly professional course, subjecting them —in many cases—to a pecuniary loss; in reality, living in advance of their time, instead of locking the wheels to its advancement, were constantly strugling to promote their calling; hoping to finally plant it, conspicuous, among the different professions, above and free from reproach, and we, as their representatives, should strive, by our individual acts, to show our gratitude for sacrifices made by them in an earlier day.

To those who have taken an interest in the progress of dentistry in Oregon, the prospect of the profession at this moment must afford supreme satisfaction. The year 1874 has brought with it success, little anticipated, and hopes beyond our most sanguine expectations. Those entertaining different opinions have done so in friendship; personalities, are well nigh extinguished, and professional jealousy seems to have lost its foothold among us; need I say, that this has resulted in great good to ourselves and patients? that each member holds his practice more securely, and while things often conspire inside, to vex, we feel at least, that we have not an enemy—just outside the door—to fight, in the shape of a professional brother.

Meanwhile, it is an encouraging fact, that the public are not uninterested spectators of our progress, and look with favor upon the earnest exertions now being made. They believe, and rightly too, that those having the advantages of a systematic education are better qualified than they who learn by intuitive skill only.

There can be no doubt that the common belief, thus expressed, is but a first acknowledgment—and consequently encouragement, to those who earnestly strive to advance themselves.

The man who is educated in general things, and is equally educated in any specialty he may follow, stands at all times, and in all places, the best chances of success. Many will say, that all this talk about "professional dignity," is not applicable to their wants, that it is their individual advancement and gain they are more particularly interested in. This is undoubtedly true of all of us, but that we may individually be benefited, we must disarm ourselves of selfishness, that good may come of, and redound to our interest. There are, however, a class, that can not see this, and no amount of gusty talk, upon the highest pressure wind-bag principle, would do them any good; they need, professionally, *to be reconstructed.* Our vocation is no longer merely mechanical, but strictly professional, and entitled to all the rank and rights, thence resulting. Dentistry in the far West, you may say, is different from dentistry in the East, that we have not the appreciative material on which to work, that our Eastern brethern have. True! Then wherefore should the labor, that rightly belongs to the united "craft," be borne by one or two representative men. The advancement made in our profession, within the last ten years, is marvelously wonderful. The contemned, and indifferently spoken of individual, then, because of his calling, is now frequently consulted by, and with, the representatives of our older, and heretofore rather patronizing sister—medicine.

But, if we are as tenacious of maintaining the respectability of our calling, stand as firmly by, and protect it as loyally,—even though she may sometimes assume to dictate, and patronize, as older members of a family sometimes do—it is only a matter postponed, when we will be heard at large, and awarded according to our just, and individual merits. As you have chosen a branch of business in which you are constantly at work for the good of others, you are dependent on what you receive from that work for your support; and who will say, when in consideration of the responsibility of that trust, and the necessity of your whole mind being engaged in it, that it should not be enough to relieve you of all anxiety on the subject of want. It should be enough to give you means to keep pace with the rapid improvements now being made, for your patrons demand it, and you are wronging them if you do not. It should be enough to clothe, feed and shelter yourself and family, at the same time provide for your wants in old age. The dentist who endeavors to get business, or establish himself in business by offering a lower rate of fees, than those about him, engaged in the same calling, *invites* his competitors to a dishonest course of action; degrades his profession, and retards its advance; consequently, injures himself and his practice.

It is unfortunate, alike, for the people and profession, that public opinion can not appreciate the difference between skillful and indifferent work. I have found almost invariably, that the difficulty in regard to price originates in the patient delaying, until they need an unexpectedly large amount of work, rather than from the dentist charging more than the operation is worth. Considering the eminent degree of perfection to which the science has attained, there is no reasonable excuse for educated people neglecting their teeth to the extent they do. While they spend their money frivolously upon health destroying luxuries, it is only when necessity compels that they consult the proper medium for relief, then cry extortion from mouths they would be ashamed to have examined.

To more graphically describe my meaning, I will quote from Moore, who, evidently, had come upon some one, whose mouth he took exception to, and you. no doubt, my professional brethren have often stumbled upon the same. The following are the lines:

"What pity blooming girl, that lips so ready for a lover,
Should not beneath their ruby casket cover,
One tooth of pearl, but like the rose beside the church-yard stone,
Be doomed to blush o'er many a mouldering bone"

This, evidently, would have been one of Moore's favorite girls, but for the disgusting exhibition of a neglected and too filthy mouth. Gentlemen! have you never met with such? and when they are presented for your consideration, is it not something of a strain upon the imagination to place our calling above the level of a scavenger? Do we make acquaintance with, hang over and inhale the poisonous gasses of a fetid breath because we love it? If not, then for *health's sake*, demand a *respectable fee*, for what *should be* respectable services rendered.

This can only be accomplished by a united effort on the part of the several members of the profession, acting in good faith, in the different communities in which they practice. No man has the right, though he possesses the power, to stigmatize or degrade any particular department of life in which he may be engaged, because he can not appreciate its importance, and thus throw or force it into the hands of charlatans or ignorant men. Concerning the late improvements in operative dentistry; the subject is so vast, covering such an extent of ground, that it is with doubt and misgivings, I undertake to write upon it. There is no subject which so interests the progressive dentist as the saving of natural teeth; it has not, however, received too much attention, for we all recognize the fact, that, in the thoroughness of this work, lies the greatest benefit we can bestow upon those, who unfortunately find it necessary to employ us. The subject is one which should be treated in sections, and demands a greater amount of attention and time than I can now devote to it. It is scarcely necessary to say, that the decay should be thoroughly removed, and that the filling be perfectly secure, too much care can not be given in shaping the cavity.

All cavities however, are not alike, either in shape or location; hence, it is necessary to classify and describe them. There seems to be but little excuse now, for an operator to slight or do what he does indifferently; and with the many improvements and valuable appliances at his command, the *public insist* that he shall do his work well or *retire from a vocation he disgraces;* this they do by with-holding patronage from him, or transfering it to others more worthy. A few people—not the masses—begin to estimate, approximating the true value of their teeth and the profession—as if grateful—have been untiring in their efforts to invent labor and pain saving appliances, whereby the work may he done to the least possible inconvenience of the patient. Very few, stop to think, how important are the natural teeth to health—we can more fully understand their merit over artificial ones, as we, *sometimes*, have to *raise the window* while repairing the latter—how miserable their lives are made by their neglect; more by a series of disorders that follow after, than their loss. We understand perfectly that the digestive organs are expected to perform their alloted task, and that only, but in the absence of teeth—or, but a "corporals guard," of what should be a "full company," remain—they have to perform a double duty, viz: masticate and digest both. This, they do until over-taxed; and worn out, tired nature gives way; then disease runs riot throughou the body, and relief is sought in medicine which is only adding insult to those already outraged organs; what may prove a temporary relief, will only hasten disease, preventing the performance of their proper functions. These, are those broken down, worn out, dyspeptic patients, that some of our medical brothers have on their hands nearly all their lives. They stand ever ready victims, a living receptacle for all the villainous compounds that may be prescribed

Should we not, to the fullest extent of our power, try to revolutionize this, and is this not our work? Much good may be accomplished, and I would recommend it, that while we strive to make *ourselves competent*, we educate our patients and the people in communities in which we practice concerning those things they seem so ignorant of, by a free and thorough distribution of pamphlets published by and under the control of our State Dental Society.

There are few dentists who do not incline to one or the other of the two departments which have been blended into our profession as one. If his inclinations lead him into the mechanical, let him stay there, that he may not dishonor himself and others by undertaking what he has no taste for and, perhaps, not the ability to perform; but let him be *thorough*, do his work well, and he will distinguish himself no matter in what department he may be engaged. If he inclines to the operative, I would repeat, after one has qualified himself, a thorough and conscientious performance of his duty, (the fee to be a secondary consideration, depending upon services rendered,) should always actuate him in his course. It is not unfrequent, that we find those operations requiring but little skill, done, with apparent disregard of consequences. Take for example, the crown eavities; I speak more particularly of these because of their considered simplicity they do not receive that attention which is absolutely necessary, and is generally given to more complicated, and difficult operations.

This work is more often found upon the molar teeth, if anything, in favor of the inferior.

The salvation of a tooth does not depend so much upon the proper introduction of gold, as the thorough preparation of the cavity, and nicely finishing the work when filled, blending the edges of the filling with the tooth, carefully dressing down and stoning the same. We find, more particularly on the inferior molars, small grooves, or fissures running at right angles across the center of the teeth, that, unless cut out to their fullest extent must necessarily involve the destruction of any work bestowed upon them; but in the superior molars, we have more frequently two depessions, each becoming the seat of disease, and only separated by a small portion of bone, and when decay has progressed far, unite both cavities in one. When separated as they sometimes are, I cut out with a chisel, and fill as one cavity. In all cases, where the teeth are of a soft and chalky nature, I use the chisel, and cut unsparingly, so long as there is a trace of frailty; indeed I may say, this is my favorite instrument in the preparation of cavities, even in preference to the engine, which I find so admirably adapted to our wants. With a sharp chisel, Varney pluggers, steel mallet, matrix,, rubber dam, and "dam" clamps........, there is but little chance of a good operator, being out-witted, under the most trying circumstances; in fact, as he becomes familiar with, and master of those extreme cases, it becomes rather a pleasure to meet and conquer them —for a consideration; and with each victory, comes new strength, also, a desire, to attempt something yet untried. I speak now only of those, who have their own improvement and the advancement of their profession dear at heart; for those only can become pre-eminently great in any department they may have chosen for their life work. There are many who imagine that unless there is a continual tramping in and out of their office they are doing nothing;—desire quantity rather than quality,—that they must wait upon a dozen or more during the day, hurrying one after another of them off, in a crazy manner, consume the time, and actually accomplish almost nothing, or "business is dull." I find my days of profit, are those devoted to only a few.

We are all liable, occasionally, to have our offices filled beyond their capacity for convenience, and at such times are apt to do things, in—what would seem to those waiting—indecent haste. There is a way, however, by which this can be avoided, and having tried them both, know whereof I speak, viz: never "block out" more work than you can comfortably perform each day, and *under no circumstances* permit yourself to be hurried in that work, even though your neighbor might get a stray patient; if you do, there is a greater chance, that you may never have the *opportunity* to refuse on account of having too much to do. Those who come because they desire you will go to no one else, unless you send them, and by the use of appointment book and cards you can easily arrange, what might otherwise involve you in trouble throughout the day.

Secure and maintain a higher standard of professional skill by doing less, and that thoroughly, and you will be more sought for and better paid.

Independence and reformation are the words by which I designate the rapid advance now being made, whose tidal wave is rolling breast high over the length and breadth of our United States, yes, we may say, is penetrating, every civilized country, the motto: protection to ourselves, and the ennoblement of our professlon.

